# Real world data on light chain cardiac amyloidosis: Still a delayed diagnosis

**DOI:** 10.3389/fonc.2022.944503

**Published:** 2022-10-06

**Authors:** Sofia Chatzileontiadou, Thomas Zegkos, Christina Frouzaki, Athanasia Apsemidou, Georgios Efthimiadis, Despoina Parcharidou, Maria Papaioannou

**Affiliations:** ^1^ Hematology Unit, 1st Department of Internal Medicine, AHEPA University Hospital, Aristotle University of Thessaloniki, Thessaloniki, Greece; ^2^ 1st Cardiology Department, AHEPA University Hospital, Aristotle University of Thessaloniki, Thessaloniki, Greece

**Keywords:** cardiac amyloidosis, light-chain amyloidosis, real world data, monoclonal gammopathy, restrictive cardiomyopathy

## Abstract

Cardiac amyloidosis (CA) represents a myocardial disorder developed by fibril deposition of a heterogeneous group of misfolding proteins. Despite being rare, a high clinical index of suspicion and novel advanced diagnostic methods seem to facilitate its early recognition. Currently nine types of cardiac amyloidosis have been described with AL and ATTR being the most common. Light chain amyloidosis (AL) is a life-threatening disease, resulting from clonal plasma cells that produce amyloidogenic light chain fragments causing organ damage including the heart. Morbidity and mortality of these patients is strongly associated with the severity of cardiac involvement. Thus, early and precise diagnosis is crucial for prompt treatment initiation. In this study, we retrospectively analyzed data of 36 consecutive patients who were diagnosed with AL amyloidosis and treated in our center over the past 15 years. Heart involvement was present in 33 (92%) of them while 76% had severe cardiac disease as of stage IIIa and IIIb, according to the Mayo2004/European staging system. Almost one third of these patients experienced an early death occurring the first five months of diagnosis. To capture everyday clinical practice, we provide details on clinical presentation, diagnostic challenges, and outcome of these patients.

## Introduction

Amyloidosis represents a heterogeneous group of misfolding protein disorders that are characterized by deposition of amyloid fibrils in various tissues and organs (1). So far, over 36 types of amyloidogenic proteins have been identified, but only nine of them can cause cardiac amyloidosis (2). Systemic immunoglobulin light chain amyloidosis (AL) and mutant or wild type transthyretin (ATTR) amyloidosis, account for more than 98% of the diagnosed cases with cardiac amyloidosis (3). Systemic AL amyloidosis remains a rare and life-threatening disease that can involve many organs including the heart and kidneys (4). Between 51% to 80% of patients with AL amyloidosis present with cardiac involvement, which is the major cause of morbidity and mortality in these patients (5, 6). Early diagnosis and identification of the amyloid type involved are crucial for the timely initiation of the appropriate therapy to prevent or delay organ failure (7). However, despite advances in diagnostic methods, the diagnosis of AL amyloidosis and/or cardiac amyloidosis is often delayed, due to the non-specificity of presenting symptoms (8). Disease awareness and a high index of suspicion are key elements for early diagnosis (7). We retrospectively analyzed data related to cardiac involvement from patients with AL amyloidosis who were diagnosed and treated at our center to capture everyday clinical practice, and provide details on clinical presentation, diagnostic challenges, and patient outcome.

## Patients and methods

We conducted a retrospective single-center study and screened fifty-two patients who were diagnosed with amyloidosis in our center over the past 15 years (from September 2006 to November 2021). Two independent reviewers analyzed the medical records of all patients diagnosed with amyloidosis at our center and demographic and clinical data were extracted into a database. Our inclusion criterion was a diagnosis of AL amyloidosis (with or without multiple myeloma) and exclusion criterion was a diagnosis of other types of amyloidosis. Collected clinical data included the symptoms at diagnosis, the type of organs involved, and the type of biopsy performed, cardiac biomarkers (NT-proBNP, troponin T-hs), laboratory and imaging data, past medical history, treatment interventions and overall survival (OS). Thirty-six (69%) patients had a diagnosis of AL amyloidosis. Overall survival was calculated from diagnosis to last follow-up or death. Survival analysis was carried out using the Kaplan–Meier method. Hematologic responses (HR) were defined according to the validated criteria for HR and organ response (9–11). Cardiac stage was defined according to the Mayo2004/European staging system (12–14). Decimals were rounded to the nearest whole number. Data extraction and processing was done in full compliance with the requirements of medical confidentiality. Data analysis was conducted with SPSS Statistics for Windows, Version 28.

## Results

### Demographics

The medical records of 52 patients diagnosed with amyloidosis were reviewed. Thirteen patients (25%) were excluded from downstream analysis, due to diagnosis of ATTR amyloidosis and 2 due to diagnosis of localized amyloidosis. One of the 37 (71%) patients diagnosed with AL amyloidosis was omitted from the analysis due to several missing data. In total, 36 (69%) patients with AL amyloidosis were considered for analysis. Our cohort included 18 (50%) males and 18 (50%) females, with a median age at diagnosis of 65 years (range, 47-83 years). Patient characteristics at diagnosis are summarized in **Table 1**.

**Table 1:** Baseline patients’ characteristics and outcome.

Characteristics	n (%)
Total number of patients	36 (100)
Median age (range), years	65 (47-83)
Sex	
Male Female	18 (50) 18 (50)
Median time from initial symptoms to diagnosis (range), months	4 (1-55)
Initial signs and symptoms	
Fatigue Weight loss Dyspnea Peripheral edema Syncope Macroglossia Periorbital purpura Peripheral neuropathy Foamy urine	23 (64) 10 (28) 18 (50) 14 (39) 4 (11) 6 (17) 9 (25) 5 (14) 10 (28)
LVEF	
≥50% <50%	25 (78) 7 (22)
Median interventricular septum thickness (range), mm	16 (11-22)
Organ Involvement Heart Kidney Liver	33 (92) 11 (31) 3 (8)
Number of organ involvement	
1 2 ≥3	6 (17) 15 (42) 15 (42)
Mayo2004/European Cardiac Stage	
II IIIa IIIb NA	4 (11) 14 (39) 11 (31) 7 (19)
Bone Marrow Plasma cells	
≤10% >10%	14 (39) 22 (61)
Serum M-Spike	25 (89)
Abnormal free light chain ratio	32 (94)
Urine M-Spike	18 (78)
Light Chain	
Kappa Lambda	11 (31) 25 (69)
Heavy Chain	
IgG IgA IgM	12 (33) 2 (6) 1 (3)
Outcome	
Dead Alive	26 (72) 10 (28)

NA, not available.

### Clinical presentation

The most common symptoms and signs at presentation were: fatigue in 23 patients (64%), dyspnea in 18 patients (50%), peripheral edema in 14 patients (39%), weight loss in 10 (28%), and foamy urine in 10 patients (28%). More specific signs, such as macroglossia and periorbital purpura, were present in 6 (17%) and 9 patients (25%), respectively. More than two thirds of patients (n=25, 69%) were referred for hematological evaluation from a cardiologist. The cause of referral (n=25, 100%) was increased wall thickness and “sparkling” appearance of the myocardium on echocardiography.

### Diagnostic approach

The median bone marrow (BM) infiltration of clonal plasma cells (PCs) for the study population was 39% (range, 3-80%). However, twenty-two patients (61%) fulfilled the diagnostic criteria for multiple myeloma with a median infiltration of BM-PCs 28% (range, 15-80%) while 14 patients had AL amyloidosis with a median infiltration of BM-PCs 9% (range, 1-10%). Serum immunofixation was positive for lambda (λ) monoclonal light chain protein in 25 patients (69%), and kappa (κ) in 11 patients (31%). The immunoglobulin heavy chain was IgG in 12 patients (33%), IgA in 2 (6%), and IgM in 1 (3%) patient. Serum free light chain ratio was abnormal in most patients (n=33, 94%). Beta2-microglobulin was abnormal in all patients with a median value of 4.25 μg/L (range, 2.55-18.9 μg/L). Renal impairment, defined as a creatinine level above 1.2 mg/dl, was detected in 15 patients (42%) of whom 9 (25%) were on hemodialysis. Urine immunofixation was performed on 23 patients (64%), and in 18 of them (78%) was positive for Bence-Jones protein.

All patients (n=36) underwent biopsy of at least one organ, including abdominal fat (n=17, 47%), bone marrow (n=36, 100%), heart (n=8, 22%), kidney (n=8, 22%), colon (n=3, 8%), liver (n=2, 6%), stomach (n=2, 6%), shoulder skin (n=1, 3%), salivary gland (n=1, 3%) and tongue (n=1, 3%). Heart involvement was present in 33 patients (92%). Among the most frequently involved organs was the kidney (n=11, 31%). In 6 patients (17%) only one organ was involved, in 15 patients (42%) two organs were involved, while 15 patients (42%) presented with multi-organ involvement (three or more). Considering the number of organ involvement, the average time from symptom onset to diagnosis was 13.67, 12.1 and 8.06 months for one, two and multi-organ involvement respectively.

The average time from symptom onset to diagnosis was 10.58 months (95% confidence interval [CI], 5.6 to 15.6), while the median time was 4 months (range, 1-55).

Even though laboratory and imaging data were highly indicative of amyloidosis, the first biopsy report for 10 individuals (28%) was negative for amyloid deposits using Congo red staining and immunohistochemistry. In the before mentioned instances, the suspected amyloidosis was confirmed by retesting the initial tissue sample in specialized centers. The misdiagnosis of AL amyloidosis was primarily owing to technical difficulties with Congo red staining.

### Cardiac features

Cardiac involvement was detected in 33 patients (92%) based on imaging techniques and confirmed with endomyocardial biopsy in 6 of them. In all patients echocardiography was performed, and echocardiograms were available for review in 32 patients (89%). Left ventricle ejection fraction (LVEF) was normal (above 50%) in 25 patients (78%), and abnormal in 7 patients (22%) with a LVEF ranging from 32 to 45%. Median interventricular septum thickness was 16 mm (range, 11-22mm). Cardiac magnetic resonance imaging (CMR) was performed in 10 patients (28%), and typical findings of cardiac amyloidosis, including subendocardial or transmural enhancement of gadolinium-based contrast, were seen in all of them. Endomyocardial biopsy was performed on 8 of these patients, and in two of them it was negative for amyloid deposits, reflecting technical laboratory issues. Median troponin T-hs concentration was 106 pg/mL (range, 0.01-570 pg/mL, normal range: <14 pg/mL), and median NT-proBNP concentration was 5,869 pg/mL (range, 262-35,000 pg/mL). Among patients with heart involvement, 25 patients (76%) had severe cardiac involvement (n=14 and n=11 with stage IIIa and IIIb, respectively), according to the Mayo2004/European staging system (12–14). Characteristics of patients with cardiac amyloidosis are summarized in **Table 2**.

**Table 2:** Patients’ with cardiac amyloidosis characteristics.

Parameters	n (%)
Total number of patients	33
Mayo2004/European Cardiac Stage	
II IIIa IIIb NA	4 (12) 14 (43) 11(33) 4 (12)
Troponin T-hs (pg/mL), median (range)	106 (0.01-570)
NT-pro-BNP (pg/mL), median (range)	5,869 (262-35,000)
Outcome	
Dead Alive	25 (76) 8 (24)
Cause of Death	
Sudden death/cardiac arrest Heart failure Not known	10 (40) 10 (40) 5 (20)

NT-pro-BNP, N-terminal pro-brain natriuretic peptide.

Troponin T-hs, Troponin T-high sensitivity; NA, not available.

### Treatment and outcome

Thirty-one patients (86%) received first-line treatment; three patients died before receiving therapy, and two patients refused any treatment. Ten patients received the combination Bortezomib/Cyclophosphamide/Dexamethasone (VCD), but two of them died shortly after starting treatment. Two patients had a very good partial response (VGPR), two had partial response (PR), one did not respond, and three patients experienced disease progression (PD). Overall, 4 patients (40%) had a PR or better response to VCD.

Ten patients were treated with Melphalan/Dexamethasone (MD). One patient achieved CR and had the longest overall survival time in our cohort (120 months); one patient achieved VGPR, and two patients achieved PR. Five of them did not respond and one patient experienced disease progression. In total, 4 patients (40%) responded to MD with PR or better.

Seven patients were treated with Bortezomib/Dexamethasone (VD), but one of them died after only 2 doses of treatment. One patient achieved CR, one achieved PR, three patients did not respond, and one patient had PD. Overall, 2 patients (33%) responded to VD with PR or better.

One patient was treated with Bortezomib/Lenalidomide/Dexamethasone (VRD) and achieved CR, while another one received Cyclophosphamide/Dexamethasone (CD) but did not respond.

Two patients received the combination of Daratumumab with either VCD or Cyclophosphamide/Thalidomide/Dexamethasone (CTD) and achieved CR and VGPR, respectively.

None of our patients underwent autologous stem cell transplantation. Second-line therapy was initiated in 11 patients (31%): four patients received Lenalidomide/Dexamethasone (RD) (two of them achieved CR and two did not respond); four patients received MD (one of them achieved VGPR, two did not respond, and one patient progressed); one patient received VCD and achieved VGPR; one patient received daratumumab-CD and achieved CR; one patient received VCD plus thalidomide and achieved PR. Treatment modalities and responses are summarized in **Table 3**.

**Table 3:** Treatment and response of patients’ with AL amyloidosis.

	Patients, n	Response, n					
		CR	VGPR	PR	NR	PD	NA
**1^st^ line**							
**MD**	10	1	1	2	5	1	
**VCD**	10		2	2	1	3	2
**VD**	7	1		1	3	1	1
**Dara-VCD**	1	1					
**Dara-CTD**	1		1				
**CD**	1				1		
**VRD**	1	1					
**2^nd^ line**							
**MD** **Prior line**	4		1 CD		2 VCD	1 VCD	
**RD** **Prior line**	4	2 VD, MD			2 MD, VCD		
**VCD** **Prior line**	1		1 MD				
**VCD-Thalidomide** **Prior line**	1			1 VD			
**Dara-CD** **Prior line**	1	1 VRD					

MD, melphalan/dexamethasone; VD, bortezomib/dexamethasone; CD, cyclophosphamide/dexamethasone.

VCD, bortezomib/cyclophosphamide/dexamethasone; RD, lenalidomide/dexamethasone.

CTD, cyclophosphamide/thalidomide/dexamethasone; VRD, bortezomib/lenalidomide/dexamethasone.

CR, complete response; VGPR, very good partial response; PR, partial response; NR, no response.

NA, not applicable.

### Survival

**Figure 1:**
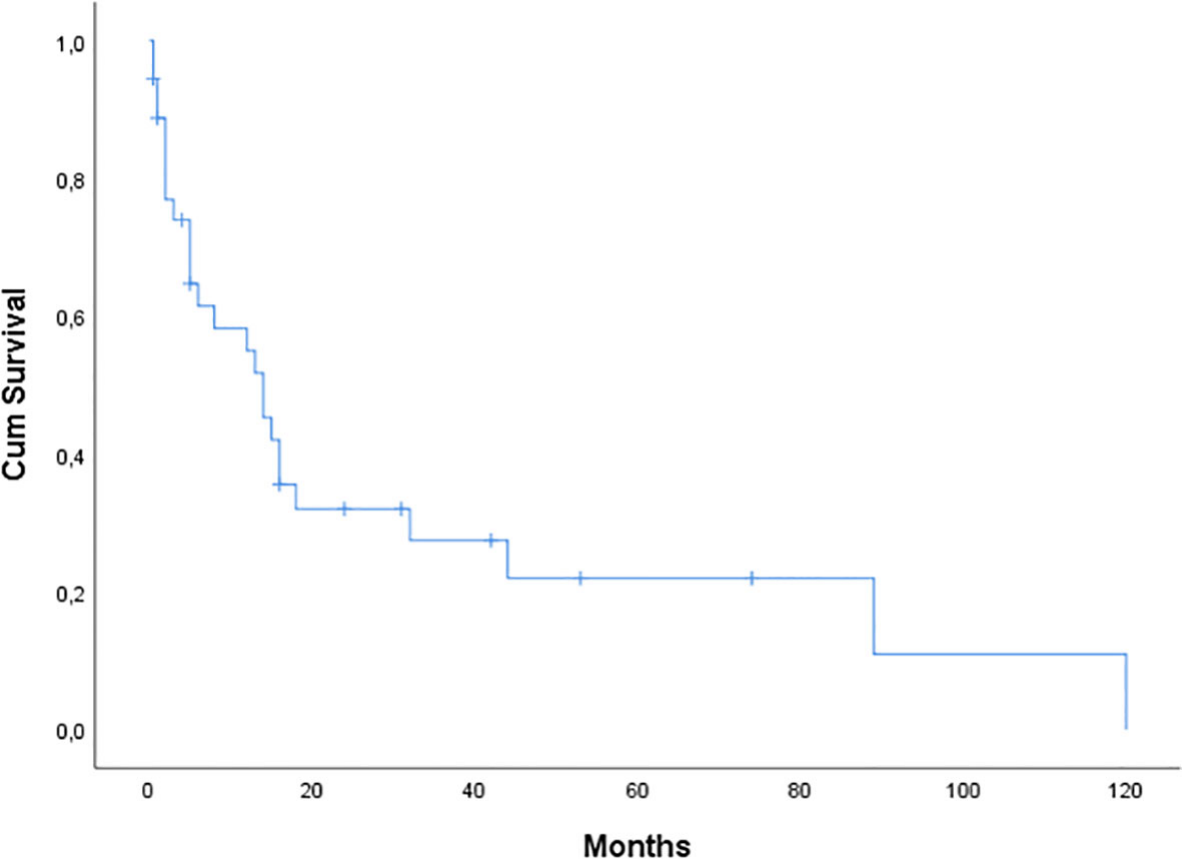
Kaplan-Meier curve of overall survival (OS) for 36 patients with AL amyloidosis seen at our center. Median OS was 14 months.

The median OS from diagnosis to last follow-up or death was 14 months (range, 0.5-120 months) (**Figure 1**). Only 8 (24%) of the 33 individuals with cardiac involvement are still alive. Four of these patients (50%) had an OS that exceeded two years. In 10 patients the cause of death was sudden death/cardiac arrest, and in 10 patients it was heart failure. The remaining five patients have unknown causes of death. Notably, 11 patients (31%) experienced early death within 5 months from diagnosis.

## Discussion

Systemic AL amyloidosis is a life-threatening disease, and early diagnosis is the most crucial goal in clinical practice. The signs and symptoms of AL amyloidosis, mostly variable and vague, are determined by the extent and number of organs involved and they are often misinterpreted and/or identified too late (9). Fatigue, weight loss, dyspnea, and peripheral edema are symptoms usually described by patients, but can be easily overlooked and their potential association with amyloidosis may be missed (8, 15). Clinical manifestations that are more indicative of amyloidosis include heart failure with preserved ejection fraction, peripheral neuropathy, nephrotic syndrome, hepatomegaly, periorbital ecchymosis, and macroglossia (8). In our study, fatigue was the most common presenting symptom (64%). Additional presenting signs and symptoms included dyspnea in 50% of cases, peripheral edema in 39%, weight loss in 28%, foamy urine in 28%, and peripheral neuropathy in 14%. Four patients (11%) experienced at least one episode of syncope. More distinctive signs of amyloidosis, such as periorbital purpura and macroglossia, were present in 25% and 17% of patients, respectively. The lack of specificity of these presenting symptoms didn’t raise initially the need to further medical investigation because they can be attributed to a variety of common and chronic conditions, such as cardiovascular disease, diabetes or cancer (16).

Organs commonly involved in AL amyloidosis include the heart in 51-80% of patients (15, 17–20), the kidneys in 58-66% (17, 18, 20), peripheral nerves in 20-34% (17, 18, 21) and the liver in 16-25% (17, 19, 21). In our cohort, heart involvement was present in 33 (92%) patients, while the kidneys were involved in 11 (31%) patients. The high proportion of patients with cardiac involvement in our cohort is related to the fact that the cardiology clinic at our hospital is a reference center for cardiac amyloidosis, which biases our referrals towards those with cardiac involvement.

In line with previous research, the average period from the onset of symptoms to diagnosis in our study was 10.28 months (95% CI, 5.6 to 15.6) (16). According to the Mayo2004/European staging system, the majority of our patients (76%) were diagnosed with severe cardiac amyloidosis (stage IIIa and IIIb). At least one tissue biopsy was performed on each of our patients, predominantly bone marrow and abdomen fat. In a few cases, cardiac, renal, liver, gastrointestinal, shoulder skin, salivary gland or tongue biopsies revealed amyloidosis. The main issue in diagnosing amyloidosis was that in more than a quarter of patients (28%), the biopsy performed was negative for amyloid deposits *via* Congo red staining, despite the likelihood of amyloidosis based on laboratory and imaging features. This represents a major diagnostic obstacle in everyday clinical practice, leading to further delays in diagnosis because more precise methods of amyloid characterization are not broadly available. Mass spectrometry, the method of choice for amyloid typing, is available only in specialized centers (3).

Cardiac involvement by amyloidosis is assessed by: (i) serum levels of cardiac troponin and NT-proBNP, (ii) cardiac imaging with Echocardiography and Cardiac Magnetic Imaging, and (iii) technetium pyrophosphate scintigraphy (PYP scan), a nuclear imaging study with >99% sensitivity and 68% specificity that is mandatory for the differentiation of AL amyloidosis with cardiac involvement from cardiac transthyretin amyloidosis. Other tracers, such as Tc-DPD (Tc-3,3-diphosphono-1,2-propanodicarboxylic acid) and Tc-HMDP (Tc-hydroxymethylene diphosphonate), are also used (3, 7). However, 30% of AL amyloidosis patients have weakly positive PYP scans and in such cases tissue biopsy can confirm the amyloid type (4).

Echocardiography was performed in all of our patients and thickening of the heart’s walls and/or granular sparkling appearance of the myocardium were frequently seen. CMR was performed in a quarter of patients (mainly those seen in recent years) and showed evidence of diffuse subendocardial late gadolinium enhancement. Tc-DPD scan was performed in patients with high suspicion for cardiac amyloidosis but normal serum free light chains and absence of monoclonal protein in serum and urine. Thirteen out of 52 patients screened were diagnosed with ATTR amyloidosis based on a positive Tc-DPD scan and normal sFLc/immunofixation.

Thirty-one (86%) patients received first-line treatment. Most patients were treated with VCD (n=10), MD (n=10), or VD (n=7), while 1 patient received VRD and 1 received CD. The regimen was selected based on the standard of care at the time of diagnosis, the patient’s presenting symptoms and their comorbidities. Patients with severe cardiac involvement were treated with caution, and sometimes under cardiac monitoring. One patient developed episodes of ventricular tachycardia and an implantable cardioverter defibrillator was placed. There is currently no conclusive proof of a survival benefit from implantable cardioverter defibrillator therapy, and randomized clinical trials are lacking. However, this should be considered in certain patients with cardiac involvement (22). Based on the recently published results of the ANDROMEDA study, we treated this patient with the combination Dara-VCD, and she responded rapidly with decrease in light chain burden and clinical improvement (23). A second patient was treated with a daratumumab-based regimen, and he attained a very good partial response. Daratumumab combination therapy is a promising first-line treatment option for these patients with an acceptable tolerability profile based on our so far, although limited, experience as well as the results from the ANDROMEDA study (23, 24). We used to treat patients with cardiac amyloidosis with doxycycline. Overall, 15 patients (45%) received doxycycline in our study. However, a phase III, randomized, open-label, multicenter study showed no benefit from the addition of doxycycline in patients with cardiac involvement (25). It is well established that the extent of heart involvement in patients with amyloidosis is the major determinant of survival (26). In our analysis, the median OS from diagnosis to last follow-up or death was 14 months (range, 0.5-120 months). As of May 2022, just 8 out of 33 patients with cardiac involvement, are still alive. Four of them (50%) with an OS of over 2 years. The main causes of death were related to cardiac involvement. Ten patients had a sudden death/cardiac arrest, and 10 patients died from heart failure. It is noteworthy that 11 patients (31%) experienced early death within 5 months from diagnosis. These findings emphasize the importance of an early diagnosis of Light Chain - Cardiac Amyloidosis. In light of the disease’s complexity and heterogeneity, it is vital that individuals with AL amyloidosis have thorough supportive care. A careful cardiac monitoring, among others, is strongly suggested, especially in the early phases of treatment, and should be performed by a multidisciplinary team comprised of different specialists knowledgeable in the management of the disease (22).

Our findings must be considered in light of certain limitations. The number of patients included in our cohort is small, and it is a retrospective study. Nevertheless, we believe that sharing data from small academic centers, like ours, is important for rare diseases such as amyloidosis, because it highlights the problems and limitations seen in everyday clinical practice, and it underscores the need for collaboration with specialized centers.

In summary, cardiac amyloidosis is a major determinant of morbidity and mortality in patients with AL amyloidosis. A high index of suspicion can facilitate early detection and prompt treatment initiation resulting in rapid decrease of the amyloidogenic light chain and potentially improved outcomes. A multidisciplinary approach is necessary for the optimal management of these patients.

## Data availability statement

The raw data supporting the conclusions of this article will be made available by the authors, without undue reservation.

## Ethics statement

Written informed consent was obtained from the individual(s) for the publication of any potentially identifiable images or data included in this article.

## Author contributions

SC collected, analyzed the data and wrote the manuscript. TZ, CF, AA, GE and DP provided, collected and analyzed data. MP designed the study, provided, analyzed the data and wrote the manuscript. All authors contributed to the article and approved the submitted version.

## Funding

The Division of Hematology of the 1^st^ Department of Internal Medicine of AHEPA University Hospital has received a research fund for the publication fee by Aristotle University of Thessaloniki Research Committee/Special Account for Research Funds (code: 84320, Title: “Molecular genetics of hematologic malignancies”).

## Conflict of interest

GE has received honoraria from Pfizer.

The remaining authors declare that the research was conducted in the absence of any commercial or financial relationships that could be construed as a potential conflict of interest.

## Publisher’s note

All claims expressed in this article are solely those of the authors and do not necessarily represent those of their affiliated organizations, or those of the publisher, the editors and the reviewers. Any product that may be evaluated in this article, or claim that may be made by its manufacturer, is not guaranteed or endorsed by the publisher.
